# Oxidative stress mechanisms and potential biomarkers of methyl acetate poisoning: a urinary metabolomics study in rat model and human occupational cohort

**DOI:** 10.3389/fmolb.2026.1801734

**Published:** 2026-04-17

**Authors:** Jiayi Lv, Jiaming Guo, Shihua Wu, Yixian Ren, Shihao Tang, Xiumei Xing, Liping Zhou

**Affiliations:** 1 Key Laboratory of Occupational Environment and Health, Guangzhou Twelfth People’s Hospital, Guangzhou, Guangdong, China; 2 School of Public Health, Sun Yat-sen University, Guangzhou, Guangdong, China

**Keywords:** cross-species metabolic disorders, metabolic reprogramming, methyl acetate poisoning, oxidative stress, potential biomarkers

## Abstract

**Background:**

Methyl acetate (MA) is a common industrial solvent that causes rapid blindness in large exposures. Its toxicologic mechanism is not fully elucidated currently. The currently used clinical marker for MA poisoning, formic acid, is unable to differentiate between MA exposure and methanol exposure, which hinders accurate diagnosis and exposure source tracing, and impairs the development and implementation of front-end preventive and control measures.

**Objective:**

This study utilized a cross-species, untargeted metabolomics approach, combining data from animal models and human cohorts, aiming to identify potential biomarkers for MA poisoning and provide new insights into its toxicological mechanisms.

**Methods:**

Subacute poisoning rat models of MA and methanol were established via gavage administration (n = 6 per group) and urine samples were collected. Meanwhile, 8 occupationally exposed MA-intoxicated patients and 10 healthy controls were enrolled, with their urine samples also being collected. All samples underwent untargeted metabolomic analysis using UPLC-QTOF/MS for comparative profiling among MA-exposed rats versus control rats, MA-exposed rats versus methanol-exposed rats, and MA-exposed patients versus healthy controls.

**Results:**

A total of 41 and 16 significantly altered metabolites were identified in MA-exposed rat models and occupationally exposed human subjects, respectively. Pathway enrichment analysis further revealed key pathways including the tricarboxylic acid (TCA) cycle, purine metabolism, glutathione metabolism, cysteine and methionine metabolism, and one-carbon metabolism, suggesting conservation of MA-induced toxic responses across species. These results indicate that MA toxicity involves not only classical TCA cycle inhibition but also close association with systemic oxidative stress. 20-carboxy-leukotrieneB_4_ (20-COOH-LTB_4_) and S-adenosylhomocysteine (SAH) were significantly elevated in the MA exposure group in both rat and human samples, but were not detected in the methanol exposure group, showing high specificity and cross-species conservation.

**Conclusion:**

This study reveals MA toxicity mechanism via oxidative stress, aids in developing therapies and enhancing MA exposure risk management. And the study identifies 20 - COOH - LTB_4_ and SAH as potential and sensitive biomarkers for MA intoxication, offering a tool for differentiating MA from methanol exposure clinically.

## 1 Introduction

MA is a green organic ester solvent with both lipophilic and hydrophilic properties. Owing to its low volatility, excellent solubility, and compliance with volatile organic compound (VOC) emission limits, MA is widely used in industrial and consumer fields such as coatings, resin processing, pharmaceuticals, and cosmetics (Huss et al., 2003; Heldreth et al., 2012). China is a major global producer and consumer of MA, with an annual production capacity exceeding one million tons (Jin, 2019). MA is also naturally released during wood processing, further expanding the exposure pathways for occupational populations (Bleich et al., 1998; Qin et al., 2021). With the expansion of its applications, the risk of MA exposure has become increasingly prominent, and poisoning incidents occur frequently. Occupational exposure is the primary route. Workers exposed to high concentrations of MA-based adhesives have developed severe symptoms including metabolic acidosis and visual impairment, with exposure levels at implicated sites far exceeding safety limits (Li et al., 2024). Multiple occupational poisoning incidents have been reported in workshops across various regions (Tang et al., 2021; Lyu et al., 2025). Long-term exposure has also been associated with malignant nasal tumors (Bleich et al., 1998). Additionally, acute exposure can cause blindness (Ogawa et al., 1988), and even accidental ingestion of small amounts in daily life can lead to adverse effects (Minns et al., 2013). Given its diverse exposure pathways, both occupational and general populations face health risks, making MA toxicity a public health issue requiring urgent attention.

Toxicokinetic studies have confirmed that MA is rapidly hydrolyzed to methanol (ME) upon entering the body. ME is further oxidized to formaldehyde and finally converted to formic acid (Kazunori et al., 1992; Heldreth et al., 2012; Nekoukar et al., 2021; Liberski et al., 2022). Both clinical case reports (Ogawa et al., 1988; Minns et al., 2013; Li et al., 2024) and animal experiments (Liesivuori et al., 1987) have verified that the accumulation of formic acid is the key mediator of toxicity, leading to metabolic acidosis and optic nerve damage. Consequently, blood and urinary levels of ME and formic acid are widely used as biomarkers for MA exposure, as with methanol exposure. Of critical importance, the misclassification between MA poisoning and methanol (ME) poisoning represents a substantial and underrecognized challenge in both clinical management and public health risk control. Clinically, MA and ME poisoning present with highly overlapping manifestations, and their core treatment regimens are largely analogous. Importantly, while such misclassification does not markedly compromise immediate therapeutic efficacy, it severely impairs accurate diagnosis and exposure source tracing. This confusion significantly obstructs the development and implementation of targeted front-end preventive and control measures specific to MA or ME exposure. Failure to accurately distinguish the true exposure source not only obscures the genuine epidemiological characteristics of MA poisoning but also limits the capacity to carry out targeted interventions for specific risk factor elimination, thereby posing a sustained threat to public health security. Compounding this dilemma, current diagnostic practice relies exclusively on blood and urinary levels of ME and formic acid as biomarkers for both forms of poisoning. These shared metabolic indicators lack the specificity to differentiate whether the toxic exposure originates from MA or ME, thereby exacerbating diagnostic ambiguity and perpetuating challenges in precise exposure attribution. Furthermore, these indicators only reflect exposure status and cannot reveal the molecular mechanisms of MA toxicity or support early warning (Kazunori et al., 1992; Nekoukar et al., 2021). Most existing research is limited to the linear metabolic pathway (MA → ME → formic acid), with insufficient investigation into how MA exposure reprograms broader metabolic networks involving carbohydrates, lipids, and amino acids. These gaps have constrained a comprehensive understanding of MA toxicity mechanisms and hindered the development of highly specific biomarkers.

This study established a MA intoxication model in Sprague-Dawley (SD) rats and performed untargeted metabolomics analysis on urine samples from the model rats. From the perspective of metabolic pathway disturbance, we explored the toxic mechanism of MA exposure, and screened potential biomarkers that could distinguish MA intoxication from methanol (ME) intoxication. We then conducted biochemical experiments to validate the key metabolites involved in dysregulated metabolic pathways. Furthermore, we performed urine metabolomics verification using an independent cohort of humans with occupational MA exposure. These findings address the limitations of conventional indicators (methanol and formic acid) that fail to discriminate MA and ME exposure, and offer novel molecular support for the early diagnosis of MA intoxication, exposure source tracing, and the refinement of health risk assessment systems.

## 2 Materials and methods

### 2.1 Chemicals and reagents

Methyl acetate (purity ≥99%) was purchased from MACKLIN Reagent Co., Ltd. (Shanghai, China). Olive oil (pharmaceutical grade) was obtained from Innochem Technology Co., Ltd. (Beijing, China). Methotrexate was purchased from AbMole Biotechnology Co., Ltd. (Shanghai, China). Methanol (LC-MS grade) was acquired from Anpel Experimental Technology Co., Ltd. (Shanghai, China). Distilled Water was supplied by Guangzhou Watson’s Food & Beverage Co., Ltd. (Guangzhou, China).

### 2.2 Animals and treatment

Thirty 10-week-old healthy male Sprague Dawley rats were obtained from the Guangdong Institute of Biotechnology (China). After 1 week of adaptive feeding, 30 rats were randomly divided into 5 groups (n = 6 per group): a control group, low-dose MA exposure group, medium-dose MA exposure group, high-dose MA exposure group, and the methanol exposure group. To model human formic acid metabolism, all rats received daily intragastric administration of methotrexate (0.3 mg/kg) for 7 days to induce a low-folate state. Following model establishment, from day 8–10, the control group received olive oil daily, while the low-, medium-, and high-dose MA exposure groups were administered MA in olive oil at 2, 3, and 4 g/kg/day, respectively. The methanol group received an aqueous methanol solution at 1.5 g/kg/day. On day 10, following the final administration of MA, methanol, or olive oil (control), rats were returned to their regular cages for routine feeding and housing. At 24 h post the final dose (i.e., on day 11), urine samples were collected manually as single spot samples from each rat immediately after voiding. The collected urine was promptly aliquoted into pre-labeled cryovials and stored at −80 °C. Following urine collection, rats were deeply anesthetized with isoflurane. Blood samples (both anticoagulated and coagulated) were collected via abdominal aortic puncture. Rats were then dissected on ice, and eyeballs were promptly harvested, rinsed with ice-cold PBS (pH 7.4), flash-frozen in liquid nitrogen, and stored at −80 °C. All procedures complied with national regulations on animal administration and were approved by the Ethics Committee of the Guangdong Institute of Biotechnology (Approval No. IACUC2025121).

### 2.3 Sample collection

Urine samples were collected from 8 patients with acute methyl acetate (MA) poisoning during the acute phase of hospitalization and 10 healthy occupationally - matched controls (Approval No. 2022104). All participants were recruited from the same workplace. Urine samples were collected from patients immediately upon hospital admission and prior to any therapeutic intervention. For both patients and controls, samples were collected during working hours (non-first-morning voids) to control for circadian variations. The samples were immediately aliquoted and stored at −80 °C until subsequent analysis.

### 2.4 Experimental methods

#### 2.4.1 Determination of methanol and formic acid concentrations

Each sample (plasma or eyeball homogenate) was derivatized by mixing with the derivatization reagent (10% sulfuric acid, 20% isopropanol, 70% pure water) at a 1:1 volume ratio. Gas chromatography-mass spectrometry (GC-MS) analysis was conducted using an Agilent 7890B GC system interfaced with an Agilent 5977B quadrupole MS detector. The system was equipped with an automatic headspace sampler and a RESTEK Rxi-624Sil capillary column (60 m × 250 μm × 1.4 μm). The injector was operated at 180 °C in split mode with a 10:1 split ratio. The column temperature was programmed as follows: initial temperature 45 °C held for 6 min, increased to 210 °C at 30 °C/min, and held for 0.5 min. The mass spectrometer was operated in electron impact (EI) mode with the ion source temperature set to 230 °C. Data were acquired in both full-scan (m/z 10–350 amu) and selected ion monitoring (SIM) modes. Characteristic ions and specific time windows were optimized for methanol, ethanol, formic acid, and acetic acid. The analytical method for determining methanol and formic acid concentrations via HSGC-MS was detailed in our earlier work (Wu S. et al., 2024).

#### 2.4.2 Pathological H&E staining

The right eyeball of each rat was rinsed with PBS and immediately fixed in a dedicated FAS eye fixative (protected from light) for 48 h. The fixed tissues were embedded in paraffin and sectioned consecutively at a thickness of 4 μm using a microtome. The sections were baked at 60 °C for 2 h to enhance adhesion. Conventional hematoxylin and eosin (H&E) staining was performed according to standard procedures. Briefly, after dewaxing and rehydration, the sections were stained with hematoxylin, differentiated, blued, and counterstained with eosin. Finally, the sections were dehydrated, cleared in xylene, and mounted with neutral gum. Staining results were examined under an optical microscope, with nuclei appearing blue and the cytoplasm red.

#### 2.4.3 Metabolomics

The frozen samples were thawed at 4 °C. An aliquot of 200 μL was then combined with 800 μL of pre-cooled methanol (−20 °C) and mixed gently. The mixture was centrifuged at 12,000 rpm for 10 min at 4 °C to precipitate proteins. A volume of 150 μL of the clear supernatant was transferred to a vial for subsequent detection. A quality control (QC) sample was generated by pooling equal aliquots (50 μL) of the supernatant from every sample. Chromatographic analysis was conducted using an Agilent 1,290 Infinity II liquid chromatography system. Separation was carried out on a Poroshell 120 EC-C18 column (3.0 × 150 mm, 2.7 μm) maintained at 40 °C. The mobile phase was composed of (A) water containing 0.1% formic acid and (B) acetonitrile, with a flow rate of 0.400 mL/min. The injection volume was 3.0 μL. The reversed-phase chromatographic separation employed the following gradient program: 0–1.5 min, 5% B; 1.5–15 min, 5% → 55% B; 15–20 min, 55% → 95% B; 20–30 min, 95% B; 30–30.1 min, 95% → 5% B; 30.1–35 min, 95% B. Mass spectrometric analysis was performed on an Agilent 6545B Q-TOF spectrometer equipped with a Dual AJS electrospray ionization (ESI) source operating in positive ion mode. The capillary voltage and fragmentation voltage were set to 3500 V and 140 V, respectively. Ion source parameters were as follows: drying gas temperature 350 °C (flow rate 8 L/min); sheath gas temperature 350 °C (flow rate 2 L/min); nebulizer pressure 40 psig. The all-ions MS/MS acquisition spanned m/z 50–1,100 at collision energies of 0, 10, and 40 eV. A quality control (QC) sample was analyzed after every 6-8 experimental samples to monitor instrument stability and reproducibility.

### 2.5 Determination of plasma TNF-α, IL-1β and MDA levels

Inflammatory cytokine levels in plasma, including TNF-α and IL-1β, were measured using ELISA kits (Rat IL-1β ELISA Kit, Cat. No. AD3032Ra, AndyGene Co., Ltd.; Rat TNF-α ELISA Kit, Cat. No. AD3238Ra, AndyGene Co., Ltd.) following the manufacturer’s protocols. Meanwhile, malondialdehyde (MDA) content in plasma was detected using ELISA kits (Rat SOD ELISA Kit, Cat. No. AD2872Ra, AndyGene Co., Ltd.) in accordance with the manufacturer’s guidelines.

### 2.6 Statistical analysis

The normality of data distribution was assessed via the Shapiro-Wilk test, and variance homogeneity via the Brown-Forsythe test. Statistical analyses were tailored to data types: continuous variables were analyzed with t-tests/ANOVA (one-way ANOVA and Bonferroni for normal/homogeneous data, Welch ANOVA and Games-Howell for normal/heterogeneous data, Kruskal–Wallis H and Dunn for non-normal data); categorical rates used Fisher’s exact test; correlations used Spearman’s rank correlation test. For the LC-QTOF-MS data processing, raw data were first processed using Agilent MassHunter Profinder software to perform initial peak detection, feature extraction, and reverse peak alignment. The resulting data matrix was processed in Mass Profiler Professional (MPP) for retention time alignment, and 75th percentile shift normalization was subsequently applied to account for potential variations in urine concentration/dilution across samples prior to statistical analysis. Strict quality control was applied to the processed data. Features were retained only if they were detected in more than 70% of the samples within at least one experimental group and demonstrated a coefficient of variation (CV) below 30% across the quality control (QC) samples.

Multivariate statistical analyses were employed to interrogate the metabolomic data. Unsupervised principal component analysis (PCA) and sample correlation analysis were first performed using MPP software to assess intrinsic data clustering and overall trends. Subsequently, supervised orthogonal partial least squares-discriminant analysis (OPLS-DA) was conducted in SIMCA to maximize group separation and identify candidate biomarkers. Differential metabolites were selected based on a fold change (FC) ≥ 2.0 and a p-value <0.05 from Student’s t-test. For metabolite identification, features were first queried against databases via IDBrowser using a mass accuracy threshold of 50 ppm. Putative identifications were then confirmed by comparing experimental MS/MS spectra against public databases (KEGG, HMDB, MassBank) with a mass tolerance of 50 ppm, using MassHunter Qualitative Analysis. Pathway enrichment analysis of the confidently identified metabolites was performed using MetaboAnalyst (version 6.0), with a p-value <0.05 deemed significant. Finally, The diagnostic efficacy of the potential biomarkers was assessed using receiver operating characteristic (ROC) curve analysis, with the area under the curve (AUC) quantifying their discriminatory power.

## 3 Results

### 3.1 Rat body weight, methanol/formic acid levels in blood and eyeballs, and retinal histological changes

**FIGURE 1:**
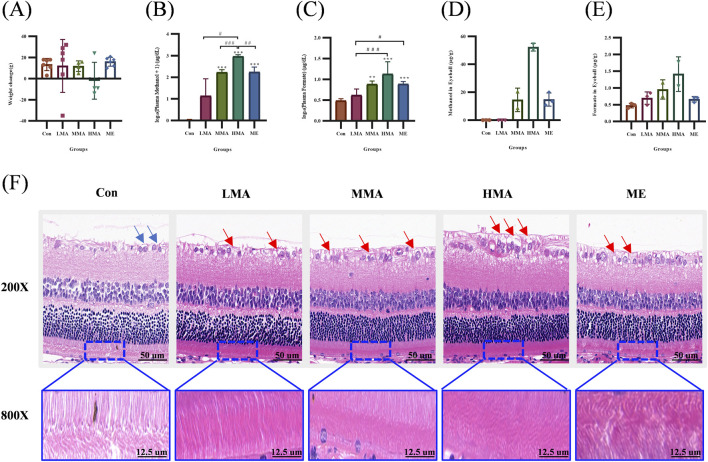
General toxicological indicators in rats after methyl acetate exposure. **(A)** Body weight change from Day 1 pre-exposure to Day 4 post-exposure. **(B)** Log-transformed plasma methanol concentration (log(methanol concentration +1)). **(C)** Log-transformed plasma formic acid concentration (log(formic acid concentration)). **(D)** Methanol concentration in eyeball tissues. **(E)** Formic acid concentration in eyeball tissues. **(F)** Histopathological analysis of retinal tissues. The first row displays a 200× view of the overall retinal structure. The second row presents an 800× magnified view of the photoreceptor layer. Black arrows: normal ganglion cells; red arrows: swollen cells with cytoplasmic vacuolation. Data are shown as mean ± SD. Sample sizes: n = 6 for Con, LMA and ME, n = 4 for MMA and HMA **(A–C)**; n = 3 for Con, LMA and ME, n = 2 for MMA and HMA **(D–E)**. *p < 0.05, **p < 0.01, ***p < 0.001, vs. Con; #p < 0.05, ##p < 0.01, ###p < 0.001, between exposure groups. Abbreviations: Con, control; LMA, low-dose methyl acetate; MMA, medium-dose methyl acetate; HMA, high-dose methyl acetate; ME, methonal.

After 3 days of MA exposure, there was no statistically significant difference in body weight gain between MA-exposed groups (low-dose LMA, medium-dose MMA, high-dose HMA) and the control group, but a decreasing trend was observed in the MA-exposed groups (Figure 1A). Plasma analysis showed that methanol concentrations in all MA-exposed groups (LMA, MMA, HMA) were significantly higher than that in the control group. Among them, the methanol concentration in the MMA group was significantly higher than that in the LMA group, while no statistically significant difference was found between the HMA and MMA groups, despite a numerical increasing trend (Figure 1B). For formic acid levels, only the MMA and HMA groups were significantly higher than the control group, with no significant difference between the LMA group and the control group (Figure 1C). Descriptive statistics only were performed for methanol and formic acid contents in eyeball tissues, with no intergroup significance testing conducted. Observational results showed that methanol content in the eyeball tissues of the MMA and HMA groups was higher than that in the control and LMA groups (Figure 1D), while formic acid content in the eyeball tissues of all MA-exposed groups was higher than that in the control group (Figure 1E). Both metabolites exhibited a clear increasing trend with the elevation of exposure dose. Additionally, histological analysis of retinal tissues via H&E staining revealed obvious abnormalities (Figure 1F). Under 200× magnification, fields of view were randomly selected for cross-group comparison to evaluate gross morphological changes, while 800× magnification revealed marked structural alterations in the retinas of MA-exposed animals compared with the intact, well-organized structure in controls. These changes were characterized by cytotoxic edema, manifested as swollen ganglion cell bodies with vacuolated cytoplasm; densely packed nuclei in the inner and outer nuclear layers with loss of intercellular gaps; and compaction of the photoreceptor inner and outer segments, which lost their normal loose organization. Notably, the methanol group (ME) exhibited similar changes to the MMA group in all above indicators.

### 3.2 Metabolomics analysis

#### 3.2.1 Total ion chromatogram (TIC) of QC samples

**FIGURE 2:**
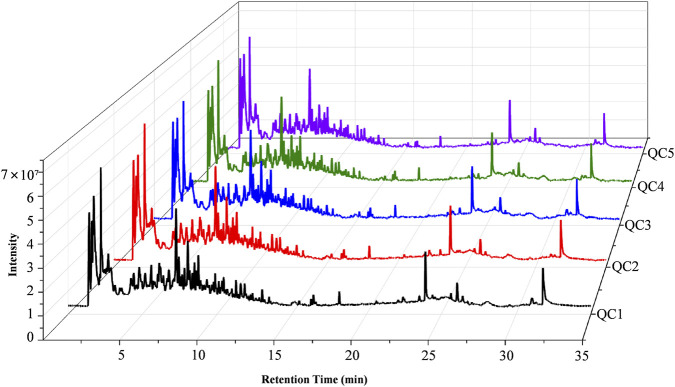
Representative TIC of QC samples.

The total ion chromatograms (TICs) of the quality control (QC) samples demonstrated highly overlapping peaks (Figure 2), indicating excellent instrumental stability and high reproducibility throughout the UPLC-QTOF analysis. To further confirm the reproducibility of the analytical method, the coefficient of variation (CV) was calculated for each detected signal across all QC samples. A large proportion of signals exhibited CV values within an acceptable range (below 30%), further supporting the good stability and reliability of the metabolomics platform. The detailed intensities and CV values of signals in QC samples are presented in Supplementary Table S1.

#### 3.2.2 Principal component analysis (PCA) and metabolic profile correlation analysis

**FIGURE 3:**
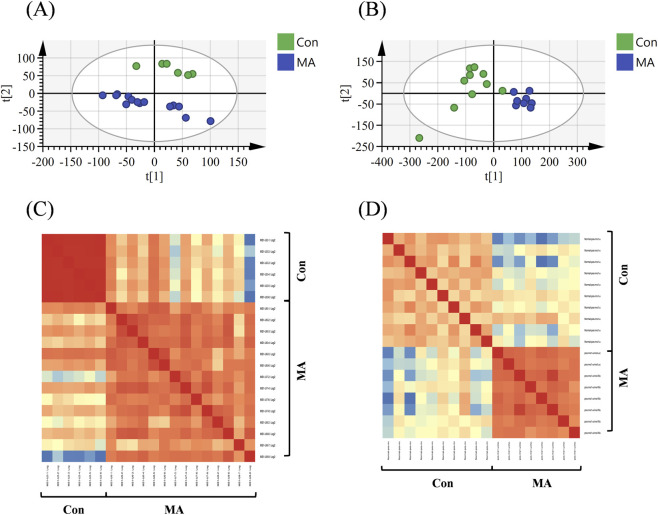
Multivariate analysis of metabolic profiles in rat and human urine samples. **(A,B)** PCA score plots derived from the urine metabolome of **(A)** rats (n (MA) = 14, n (Con) = 6) and **(B)** humans (n (MA) = 8, n (Con) = 10). **(C,D)** Pearson correlation heatmaps for **(C)** rat and **(D)** human samples. The color gradient from blue (0, no correlation) to red (1, strong positive correlation) represents the magnitude of the correlation coefficient.

Although individual variations resulted in some dispersion within groups, unsupervised PCA clearly distinguished the MA group from the control group in both rat (Figure 3A) and human (Figure 3B) samples. Furthermore, Pearson correlation coefficients were calculated to assess the similarity of metabolic profiles across all samples in both species and visualized using heatmaps (Figures 3C,D). High correlations were observed among samples within each group (MA or control) in both species. In contrast, low inter-group correlations highlighted a clear distinction in metabolic profiles between MA-exposed and control conditions. These correlation-based findings are consistent with the PCA results across species, confirming that MA exposure induces significant and consistent alterations in metabolic profiles in both rats and humans. This indicates that the metabolic remodeling effect of MA is conserved across species.

#### 3.2.3 Orthogonal partial least squares discriminant analysis (OPLS-DA) and univariate analysis

**FIGURE 4:**
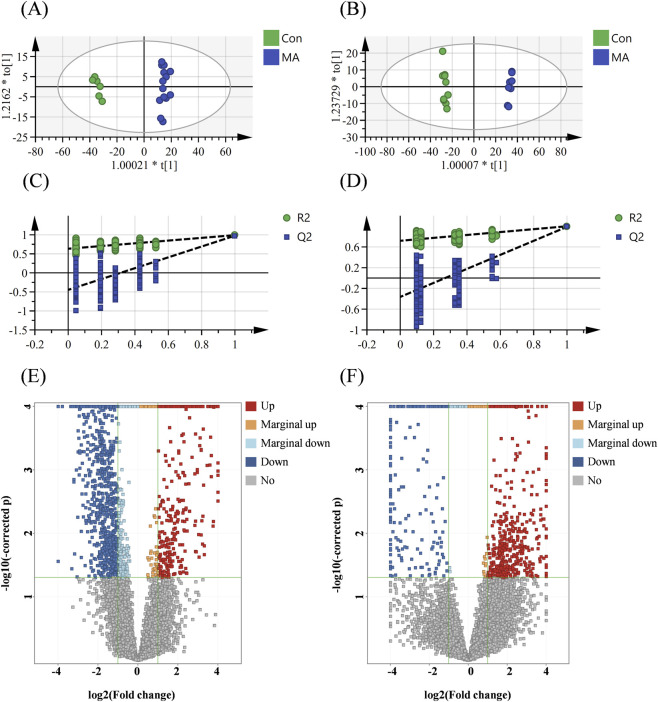
Multivariate analysis and differential metabolite screening in rat and human urine metabolomes following MA exposure. **(A,B)** OPLS-DA score plots demonstrating distinct separation between the control and MA-exposed groups in **(A)** rats (n (MA) = 14, n (Con) = 6) and **(B)** humans (n (MA) = 8, n (Con) = 10). **(C,D)** Permutation test results (200 permutations) validating the OPLS-DA models for **(C)** rat and **(D)** human data. The original model’s actual values (right points) exceed all permuted *R* ^2^ and Q^2^ values (left), confirming model robustness without overfitting. **(E,F)** Volcano plots visualizing significantly altered metabolites in **(E)** rat and **(F)** human samples. Metabolites are color-coded based on fold change (FC) and statistical significance (p < 0.05): red (significantly upregulated, FC ≥ 2.0), blue (significantly downregulated, FC ≤ 0.5), orange (marginally upregulated, 1.0 < FC < 2.0), light blue (marginally downregulated, 0.5 < FC < 1.0), and gray (non-significant, p ≥ 0.05).

To maximize the discrimination between the MA group and control group, based on metabolic profiles, orthogonal projections to latent structures-discriminant analysis (OPLS-DA) models were established. The resulting models clearly separated the MA-exposed group from the control group in both rat (Figure 4A) and human (Figure 4B) samples. To validate model reliability, 200 permutation tests were performed (Figures 4C,D). The validation results showed a negative y-intercept of the Q2 regression line, and all permuted Q2 values (left) were lower than the original point (right), confirming model robustness without overfitting. These validated models were subsequently used for differential metabolite screening.

Based on p-values from univariate t-tests and fold changes, a total of 1,602 (rats) and 815 (humans) significantly differential metabolites were screened using the criteria “p < 0.05, FC ≥ 2.0”. The distribution of these differential metabolites was visualized via volcano plots (Figures 4E,F). Further identification via database matching (HMDB, KEGG, MassBank) and MS/MS fragmentation analysis yielded 41 (rats) and 16 (humans) differential metabolites, with 10 metabolites listed for each group in Table 1 and the remaining ones provided in Supplementary Table S2.

**TABLE 1:** Differential metabolites identified in rats and humans following methyl acetate exposure.

Metabolite of rat		
Metabolite	KEGG ID	Log2 FC
3-Sulfinylpyruvate	C05527	4.00
5-Oxoproline	C01879	4.00
Succinate	C00042	4.00
Methylglyoxal	C00546	4.00
Dihydroxyfumarate	C00975	4.00
5-Aminolevulinate	C00430	−2.24
L-homoserine	C00263	−2.11
L-Glutamine	C00064	−1.86
D-Serine	C00740	−1.81
L-Cysteine	C00097	−1.70

Metabolite of human		
Metabolite	KEGG ID	Log2 FC
Cys-gly	C01419	4.49
Homocitrate	C01251	4.00
Sarcosine	C00213	4.00
15-Keto-prostaglandin I2	C04835	4.00
Dehydroalanine	C02218	4.00
S-adenosyl-L-homocysteine	C00021	2.51
20-COOH-leukotriene B4	C05950	2.26
2-Oxobutanoate	C00109	1.49
2,8-Dihydroxyadenine	C22500	1.33
Creatine	C00300	−1.98
L-cystathionine	C02291	−4.52

#### 3.2.4 Key differential metabolic pathways perturbed by MA exposure

To systematically characterize the metabolic alterations induced by MA exposure, KEGG pathway enrichment analysis was performed on differential metabolites in rat and human urine. The results revealed that MA-induced toxicity led to highly consistent disruptions in metabolic pathways between the two species (Table 2).

**TABLE 2:** Metabolic pathways disturbed by methyl acetate exposure in rats and humans.

Metabolite of rat				
Pathway name	Hit	Raw p	FDR	Impact
Purine metabolism	7/71	4.3258E-05	0.0025528	0.05548
CystEine and methionine metabolism	5/33	8.2461E-05	0.0025528	0.1396
Glycine, serine and threonine metabolism	5/34	9.5731E-05	0.0025528	0.28464
One carbon pool by folate	4/26	0.00044669	0.0089337	0.07357
Pyruvate metabolism	3/23	0.0041831	0.06693	0.10512
Glutathione metabolism	3/28	0.0073708	0.084238	0.09925
Alanine, aspartate and glutamate metabolism	3/28	0.0073708	0.084238	0.33734
Glyoxylate and dicarboxylate metabolism	3/32	0.010735	0.10735	0.11333
Arginine biosynthesis	2/14	0.017246	0.1533	0
Pantothenate and CoA biosynthesis	2/20	0.034115	0.24811	0
Citrate cycle (TCA cycle)	2/20	0.034115	0.24811	0.08276

Metabolite of human				
Pathway name	Hit	Raw p	FDR	Impact
Cysteine and methionine metabolism	4/33	5.0962E-05	0.0023018	0.26496
Glycine, serine and threonine metabolism	4/34	5.7546E-05	0.0023018	0.08874
One carbon pool by folate	3/26	0.00063557	0.016948	0.1504
Purine metabolism	3/71	0.011704	0.23409	0.06814
Glutathione metabolism	2/28	0.015694	0.2511	0.06389

**FIGURE 5:**
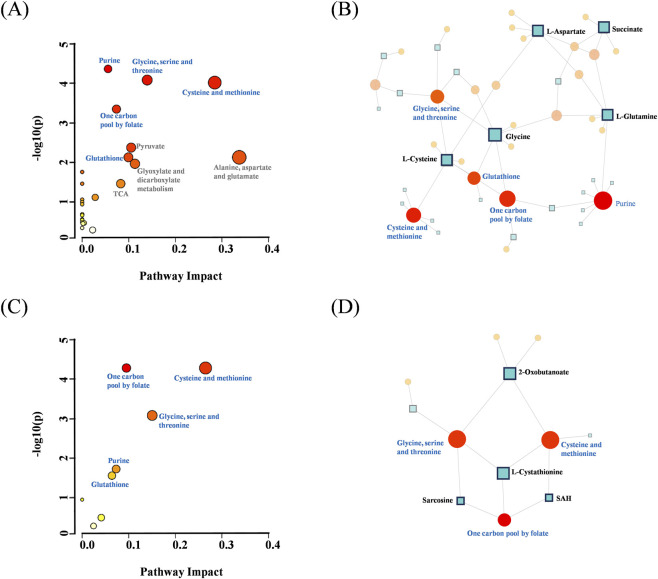
**(A,C)** Significantly disturbed metabolic pathways in **(A)** rats (n (MA) = 14, n (Con) = 6) and **(C)** humans (n (MA) = 8, n (Con) = 10) after methyl acetate exposure. Bubble size represents the impact value derived from topological analysis, while color indicates the enrichment significance (-log_10_ (p-value)). **(B,D)** Pathway interaction networks for **(B)** rats and **(D)** humans. Node size and color scheme are consistent with panels **(A)** and **(C)**. Small square nodes denote shared metabolites, and their size is proportional to the number of connected metabolic pathways (larger squares represent more pathway connections). Connecting lines represent metabolite-mediated interactions between pathways, illustrating the integrity and coordination of the metabolic network response.

In the rat model, 11 significantly enriched pathways were screened (p < 0.05), among which purine metabolism, cysteine and methionine metabolism, glycine, serine and threonine metabolism, and one-carbon pool by folate exhibited the most pronounced enrichment significance (Figure 5A). In human samples, the same four core pathways were also significantly enriched, along with pathways strongly associated with oxidative stress, such as glutathione metabolism (Figure 5C). Notably, although the false discovery rate (FDR)-corrected p-values for some pathways did not meet the stringent threshold of <0.05, these pathways—including pyruvate metabolism and the citrate cycle (TCA cycle) in rats—are biologically closely related to the core perturbed pathways, collectively forming an integrated metabolic response network. Their uncorrected p-values (p < 0.05) still provide strong evidence of biological relevance. To visualize functional interactions among these pathways, metabolite pathway interaction networks were constructed for rats (Figure 5B) and humans (Figure 5D). Both networks clearly demonstrated that the consistently enriched core pathways were closely interconnected through key differential metabolites (e.g., glycine, serine), forming a conserved functional module. This high degree of cross-species consistency strongly supports the conclusion that MA exerts its toxic effects by disrupting this core metabolic network.

#### 3.2.5 Screening of potential biomarkers for methyl acetate intoxication

**FIGURE 6:**
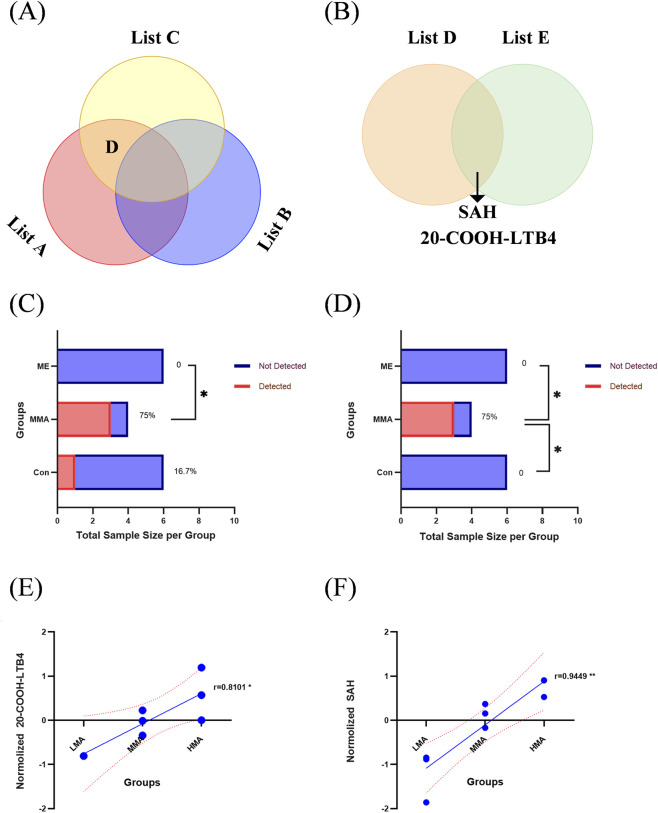
Stepwise screening and validation of cross-species core biomarkers for MA poisoning. **(A)** Three-group Venn diagram showing intersections of metabolite lists from comparisons: MMA vs. Con (list A), ME vs. Con (list B), and MMA vs. ME (list C). List D (rat MA candidates) was obtained from the intersection of A and C, excluding B-overlapping metabolites. **(B)** Two-group Venn diagram identifying the final cross-species core biomarkers (20-COOH-LTB_4_ and SAH) from the overlap of rat list D and human list E (MA vs. Con). **(C,D)** Detection rate of **(C)** 20-COOH-LTB_4_ and **(D)** SAH in Con (n = 6), MMA (n = 4), and ME (n = 6) groups. **(E,F)** Spearman’s correlation analysis showing positive associations of **(E)** 20-COOH-LTB_4_ and **(F)** SAH levels with MA doses (LMA, n = 6; MMA, n = 4; HMA, n = 4), with correlation coefficients of 0.8101 and 0.9449, respectively. Relative levels of 20-COOH-LTB4 **(E)** and SAH **(F)** were calculated from detected intensities only without missing value imputation, followed by median normalization for inter-group comparison.

A multi-step screening strategy was used to identify MA-potential biomarkers. Differential metabolite lists were generated from pairwise comparisons among the rat medium-dose MA, normal control, and methanol control groups (a methanol dose equivalent to the medium-dose MA on a molar basis), designated as list A, list B, and list C (Figure 6A). The intersection of list A and list C, excluding metabolites overlapping with list B, produced list D, representing candidates related to MA exposure rather than methanol exposure (Figure 6A). Finally, the intersection of list D with list E, which contained differential metabolites from the human cohort, selected biomarkers with cross-species conservation (Figure 6B). This process identified 20-COOH-LTB4 and SAH as the core biomarkers, corresponding to the intersecting area in Figure 6B.

20-COOH-LTB4 and SAH showed significant specific distribution patterns in SD rat Con, MMA, and ME. Both metabolites showed extremely low detection rates in the Con and ME groups but were significantly elevated in the MMA group. Further intergroup analysis revealed that the detection rate of SAH (Figure 6D) was significantly higher in the MMA group compared with both the Con and ME groups, confirming its potential association with MA exposure. For 20-COOH-LTB4 (Figure 6C), the detection rate in the MMA group was significantly higher than in the ME group. Although no statistically significant difference was observed compared with the Con group—only an upward trend was noted—this may be attributed to the limited sample size in the present study. Spearman’s rank correlation analysis demonstrated that the detection levels of 20-COOH-LTB4 (Figure 6E) and SAH (Figure 6F) were significantly and positively correlated with MA exposure dose. The doses included low dose (LMA), medium dose (MMA), and high dose (HMA). The correlation coefficients reached 0.8101 and 0.9449 respectively. This confirms a clear dose-dependent accumulation of both metabolites, supporting their potential as biomarkers for indicating exposure intensity.

**FIGURE 7:**
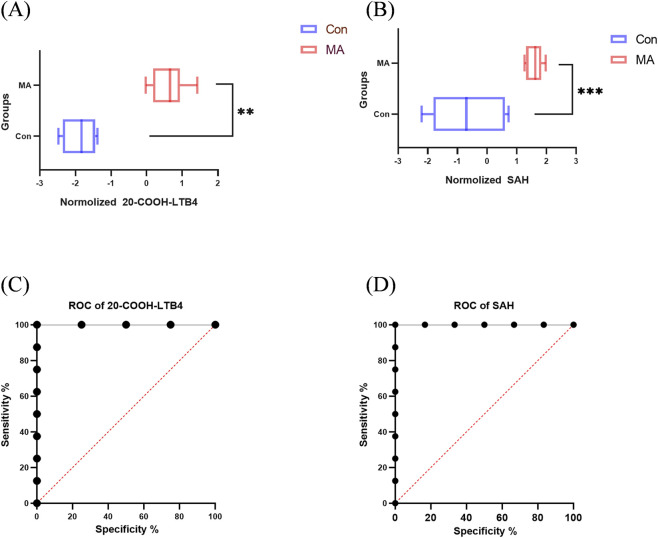
Distribution comparisons and receiver operating characteristic (ROC) curve analyses of 20-COOH-LTB4 and SAH in human normal control and MA exposure groups. **(A,B)** Detection levels of **(A)** 20-COOH-LTB4 and **(B)** SAH (n (MA) = 8, n (Con) = 10). Both metabolites were detected only in partial control samples but in all MA-exposed samples, with significantly lower levels in the control group. **(C,D)** ROC curves for distinguishing controls from MA-exposed subjects for **(C)** 20-COOH-LTB4 and **(D)** SAH. The area under the curve (AUC) values of both metabolites reached 1.00.

In human samples, 20-COOH-LTB4 and SAH also displayed specific distribution patterns. Both metabolites were detected only in a subset of normal controls, but were present in all samples from the MA-exposed group, with detection levels in controls being significantly lower than those in the exposed group (Figures 7A,B). Receiver Operating Characteristic (ROC) curve analysis indicated that the area under the curve (AUC) of both metabolites for distinguishing normal controls from MA-exposed subjects in the human population reached 1.00 in distinguishing normal controls from MA-exposed individuals (Figures 7C,D), indicating excellent discriminatory performance. This ideal result arises from the complete separation and lack of overlap in metabolite levels between the two groups. However, given the relatively small human sample size in this study, this promising diagnostic performance should be interpreted with caution. Further validation in a larger and multi-center cohort will be required in future research.

### 3.3 The effect of MA intervention on IL-1β, TNF-α, and MDA levels in rat plasma

**FIGURE 8:**
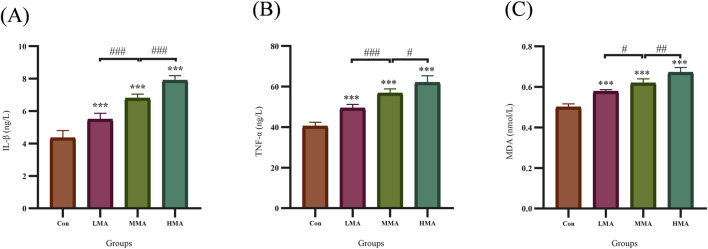
Plasma levels of IL-1β, TNF-α and MDA in rats from each group. **(A)** Plasma IL-1β level; **(B)** Plasma TNF-α level; **(C)** Plasma MDA level. Data are presented as mean ± SD. *P < 0.05 vs. Con group; #P < 0.05 for comparisons among LMA, MMA and HMA groups.

**FIGURE 9:**
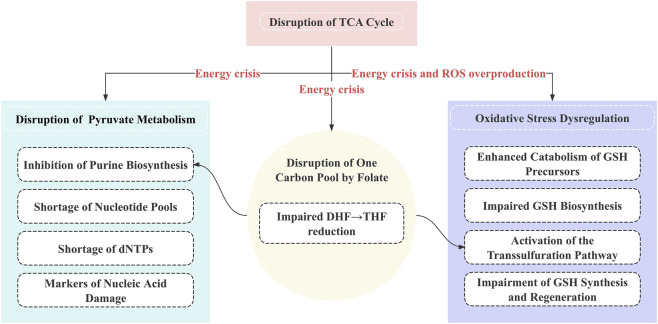
Schematic overview of key metabolic disturbance modules induced by MA. Colored blocks represent the four key metabolic disturbance modules triggered by MA exposure: disruption of the TCA cycle, pyruvate metabolism, folate-mediated one-carbon pool, and oxidative stress imbalance. Internal rectangular boxes illustrate the specific abnormal events within each module. Arrows indicate crosstalk among these modules, mediated by energy crisis, ROS overproduction, and inadequate one-carbon unit supply. These interconnected modules collectively drive the systematic metabolic disorder induced by MA.

The plasma levels of IL-1β, TNF-α and MDA in each group were measured by ELISA, and the results are presented in Figure 9. Compared with the Con group, the concentrations of plasma IL-1β (Figure 8A), TNF-α (Figure 8B) and MDA (Figure 8C) were all significantly increased in the LMA, MMA and HMA groups with statistically significant differences. Moreover, all three indicators showed an obvious dose-dependent upward trend with the escalation of MA dosage, and the most pronounced upregulation was observed in the HMA group.

## 4 Discussion

### 4.1 Successful establishment of the rat model

The successful establishment of a MA poisoning model in this study was confirmed by the markedly elevated concentrations of methanol and formic acid detected in the plasma and ocular tissues of the rats, demonstrating the absorption, metabolic conversion, and subsequent accumulation of the MA in target organs. Furthermore, this systemic exposure was corroborated by significant pathological damage in the sensitive retinal tissue, typically observed as cytotoxic edema. Together, these findings form a complete chain of evidence for the *in vivo* toxicity of MA.

### 4.2 Systemic metabolic perturbations induced by MA exposure

In this study, unbiased metabolomic analysis was performed to compare metabolic profiles between MA-exposed groups (MA group) and control groups (Con group) in both rats and humans. Specifically, 41 differential metabolites were identified in the rat model (MA group vs. Con group), and 16 differential metabolites were screened in the human cohort (MA group vs. Con group), with detailed information presented in Table 1. These differential metabolites were then subjected to KEGG pathway enrichment analysis, which revealed that metabolic disturbances induced by MA exposure (relative to the blank control) were concentrated in 11 pathways in rats and 5 pathways in humans, as summarized in Table 2. We found that all five human pathways (purine metabolism, glutathione metabolism, cysteine and methionine metabolism, glycine/serine/threonine metabolism, and one-carbon metabolism) exactly overlapped with the core disturbances in rats. This high degree of conservation strongly suggests that these five pathways represent the core metabolic network targeted by MA toxicity. The more extensive pathway disturbances in the rat model may reflect higher metabolic sensitivity or an earlier toxic response, whereas the human data pinpoint the indispensable, common pathophysiological core of the mechanism. The following discussion will focus on the four key metabolic disturbances associated with the five core pathways, and their integrated mechanistic relationships are illustrated in Figure 9. Detailed changes in the corresponding differential metabolites are shown in Supplementary Figure S1.

#### 4.2.1 Energy crisis and purine metabolism disruption

Formic acid, a metabolite of MA, inhibits cytochrome c oxidase (Liesivuori et al., 1987; Liesivuori and Savolainen, 1991; Xiao and Zhu, 2025), leading to mitochondrial dysfunction and ATP depletion, which in turn disrupts purine metabolism. In the rat model, the accumulation of tricarboxylic acid (TCA) cycle intermediates (succinic acid and cis-aconitic acid) along with increased methylglyoxal production confirms the concomitant dysregulation of mitochondrial and cytoplasmic metabolism (Kerr et al., 2000; Martínez-Reyes and Chandel, 2020). Furthermore, abnormal regulation of adenosine, adenine, and hypoxanthine in purine metabolism, as well as the accumulation of 5-aminoimidazole-4-carboxamide ribonucleotide (AICAR) and succinyladenosine monophosphate (SAICAR) in the human cohort, indicate that MA interferes with both the purine salvage pathway and *de novo* synthesis (Raivio and Andersson, 1982; Plagemann et al., 1985; Decking et al., 1997; Boza et al., 2000; Park and Gupta, 2008; Bertrand and Benoît, 2012; Hove-Jensen et al., 2017; Zu et al., 2025). Together, these alterations constitute a core pathological basis for the ensuing energy crisis.

#### 4.2.2 Amplification of oxidative stress

MA exposure induces cross-species disruption of the oxidative defense system. In the rat model, downregulation of cysteine and glycine leads to insufficient substrates for glutathione (GSH) synthesis, while decreased ophthalmate (OPH) and its precursors impair the antioxidant network (Chapple, 1996; Schafer and Buettner, 2001; Ito et al., 2018; Schomakers et al., 2024). In both rats and humans, the accumulation of S-adenosylhomocysteine (SAH) activates the transsulfuration pathway, which prioritizes GSH production to counteract oxidative stress (Finkelstein, 2007; Sbodio et al., 2019). Additionally, 5-oxoproline, commonly upregulated in both species, serves not only as a marker of impaired GSH synthesis and regeneration but is also closely linked to ATP depletion (Werf et al., 1974; Jackson et al., 1987; Mayatepek, 1999; Bains and Bains, 2015). In rats, elevated 8-hydroxyadenine provides direct evidence of DNA/RNA oxidative damage (Guo et al., 2020). Collectively, these alterations form a self-sustaining vicious cycle of oxidative stress triggered by MA exposure.

#### 4.2.3 Disruption of one-carbon metabolism

In the rat model, the upregulation of dihydrofolate (DHF) indicates impaired dihydrofolate reductase (DHFR) function (Lan et al., 2018), leading to insufficient production of tetrahydrofolate (THF) and a consequent deficiency of its activated derivatives, including 5,10-methylenetetrahydrofolate, 10-formyltetrahydrofolate, and 5-methyltetrahydrofolate (Douglas, 1987; Lewin et al., 2017; Webb et al., 2019; Petrova et al., 2023). This deficiency disrupts key downstream processes, such as purine synthesis and the conversion of SAH to SAM, positioning one-carbon metabolism as a central hub linking the dysregulation of energy metabolism and oxidative stress.

#### 4.2.4 Cross-species specificity in metabolic responses to MA exposure

Notably, although MA exposure consistently enriches core pathways including purine metabolism and glutathione metabolism in both rats and humans, significant species-specific differences exist in the differential metabolites within these pathways and their variation patterns. Details of these differences are provided in Table 1 and Supplementary Table S2. Taking purine metabolism as an example, rats harbor 7 differential metabolites such as L-glutamine, adenosine, and deoxyadenosine monophosphate (dAMP), while humans have 4 differential metabolites including 1-(5′-phosphoribosyl)-5-amino-4-imidazolecarboxamide and cysteinylglycine (Cys-Gly). Moreover, obvious divergence is observed in the direction and magnitude of changes in these metabolites between the two species.

Such cross-species differences have been well-documented in toxicological metabolomics studies and can be explained by the following interrelated factors. First is the difference in metabolic rate. According to the allometric scaling law (West et al., 1997), the mass-specific metabolic rate of rats is 5–10 times higher than that of humans, resulting in faster metabolite turnover. This directly leads to differentiated accumulation and clearance kinetic characteristics of purine metabolites after MA exposure. Second is the difference in exposure patterns. Rats undergo standardized experimental exposure, while human exposure features greater complexity. This inconsistency directly drives species-specific differences in metabolite profiles (Johnson et al., 2012). Third is the inherent physiological and biochemical differences. Species-specific variations in the activity of key purine metabolism enzymes (e.g., purine nucleoside phosphorylase, adenosine deaminase) alter pathway flux and metabolite output (Tavenier et al., 1995). Meanwhile, differences in tissue-specific metabolite distribution (Stein et al., 1988), glutathione-dependent detoxification capacity (e.g., species-specific alteration of Cys-Gly in humans but not in rats), and regulation of metabolic enzyme expression by interspecies epigenetic differences further exacerbate this phenomenon (Adedeji, 2025).

Despite differences in individual metabolites, the conservation of core metabolic pathway enrichment confirms the effectiveness of the rat model in simulating MA-induced core metabolic disorders. The identified species-specific metabolites also suggest that caution should be exercised when extrapolating animal experimental data to humans, providing valuable references for future translational research.

### 4.3 Potential biomarkers for MA exposure and their mechanistic implications

To identify biomarkers that can more specifically characterize MA poisoning and distinguish it from methanol poisoning, we performed additional screening incorporating the methanol (ME) group on the basis of comparative analysis between the MA group and the control (Con) group. Following this integrated screening approach, two metabolites were identified as particularly promising indicators of MA exposure: 20-COOH-LTB_4_ and SAH. 20-COOH-LTB_4_ and SAH were significantly elevated only in the MMA group of rats and showed strong positive correlations with MA exposure dose. In humans, both metabolites perfectly distinguished exposed subjects from controls with AUC values of 1.00 in ROC analysis. However, this result should be interpreted with caution due to the relatively small sample size (see Limitations). No obvious increase was observed in the ME group, suggesting their potential as potential biomarkers for differentiating MA poisoning from methanol poisoning.

The accumulation of SAH inhibits methylation reactions (Isakovic et al., 2009) and thereby mediates the synergistic dysregulation of multiple metabolic pathways. As a core intermediate of the methionine cycle, the specific accumulation of SAH directly results from the cross-talk disruption among these pathways induced by MA exposure. First, MA exposure impairs the function of dihydrofolate reductase (DHFR), a key enzyme in one-carbon metabolism. This leads to insufficient production of tetrahydrofolate (THF), and consequently to a deficiency of its activated derivative, 5-methyl-THF (Lan et al., 2018; Webb et al., 2019). Since 5-methyl-THF serves as the essential one-carbon donor for the conversion of SAH to S-adenosylmethionine (SAM), its shortage directly blocks the reversible SAH→SAM reaction, resulting in continuous SAH accumulation (Webb et al., 2019). Second, oxidative stress induced by MA exposure depletes substrates for glutathione (GSH) synthesis substrates (cysteine, glycine) (Chapple, 1996). To compensate for antioxidant capacity, cells activate the transsulfuration pathway. SAH, which initially accumulates due to impaired metabolism, is a potent activator of cystathionine-β-synthase (CBS) (Finkelstein, 2007). Elevated SAH levels thus enhance CBS activity, promoting the conversion of homocysteine (Hcy) to cysteine to meet GSH synthesis demand (Sbodio et al., 2019). Excessive activation of transsulfuration accelerates Hcy consumption, and this substrate-depletion signal drives increased metabolic flux through the methionine cycle, ultimately amplifying SAH production (Finkelstein, 2007; Sbodio et al., 2019). Finally, ATP depletion exacerbates SAH accumulation. Formic acid, a metabolite of MA, inhibits cytochrome c oxidase (Liesivuori et al., 1987; Liesivuori and Savolainen, 1991; Xiao and Zhu, 2025), causing mitochondrial dysfunction and systemic ATP depletion. Since the metabolic clearance of SAH depends on ATP supply, ATP deficiency further impairs SAH metabolism, forming a vicious cycle of ATP depletion → reduced SAH clearance → aggravated accumulation (Werf et al., 1974; Jackson et al., 1987). In summary, SAH accumulation is not caused by a single pathway disorder, but represents a core node of the cross-talk among the methionine cycle, one-carbon metabolism, and oxidative stress pathways induced by MA. Its dose-dependent increase directly reflects the severity of MA intoxication.

These metabolomic observations were further validated by targeted ELISA measurements of plasma inflammatory and oxidative stress markers. Compared with the Con group, plasma levels of IL-1β, TNF-α and MDA were significantly elevated in LMA, MMA and HMA groups, and all exhibited a clear dose-dependent increasing trend. The upregulation of IL-1β and TNF-α provided direct phenotypic evidence for systemic inflammatory activation (Satheesan et al., 2024; Meier et al., 2025), which is closely associated with the metabolic disorders of SAH and 20-COOH-LTB_4_ identified in our untargeted metabolomic analysis. Meanwhile, the increased MDA level directly confirmed enhanced lipid peroxidation and oxidative stress injury (Sieminski et al., 2023). Collectively, these experimental data strongly support that oxidative stress and inflammatory responses are key pathological processes triggered by MA, and establish a solid mechanistic link between the identified metabolic alterations and corresponding phenotypic changes.

20-COOH-LTB_4_, a natural antagonist and terminal metabolite of leukotriene B4 (LTB4), was upregulated following MA exposure, indicating activation of the upstream LTB4-driven pro-inflammatory pathway (Archambault et al., 2019). In the present study, 20-COOH-LTB_4_ showed dose-dependent accumulation. Together with the observed global oxidative stress signature, we tentatively infer that this accumulation results from both increased LTB4 synthesis driven by oxidative stress and decreased clearance due to glutathione (GSH) depletion. Formic acid, a metabolite of MA, inhibits mitochondrial cytochrome c oxidase (Liesivuori et al., 1987; Liesivuori and Savolainen, 1991; Xiao and Zhu, 2025), leading to electron transport chain impairment and excessive reactive oxygen species (ROS) production. This is consistent with the strong oxidative stress state identified in this study, reflected by elevated 8-hydroxyadenine (a marker of DNA oxidative damage) and accumulated 3-sulfopyruvate (evidence of amino acid oxidative damage) (Giles and Jacob, 2002; Guo et al., 2020), as well as findings from toxicological studies demonstrating that mitochondrial dysfunction-mediated ROS overproduction is a conserved toxic response to xenobiotic exposure (Zhang et al., 2025; Gong et al., 2026). According to established literature, excessive ROS can activate phospholipase A2 (PLA2), which catalyzes the hydrolysis of membrane phospholipids to release arachidonic acid (AA)—a core precursor for inflammatory mediator synthesis (Wan et al., 2017; Pecchillo Cimmino et al., 2024). AA is then specifically metabolized via the 5-lipoxygenase (5-LOX) pathway to generate LTB4 (Wan et al., 2017; Heo et al., 2021; Pecchillo Cimmino et al., 2024). Thus, we speculate that the pronounced oxidative stress in this system may drive enhanced LTB4 synthesis through this canonical pathway, thereby increasing the production of its terminal metabolite, 20-COOH-LTB_4_. This is consistent with the broader paradigm that oxidative stress is a key upstream trigger of pro-inflammatory mediator production (Zhang et al., 2025; Gong et al., 2026). Concurrently, the depletion of GSH synthesis substrates (cysteine and glycine) observed in this study (Chapple, 1996) weakens the antioxidant system’s capacity to clear inflammation-related active mediators (Pecchillo Cimmino et al., 2024), amplifying activation of the leukotriene metabolic pathway. Notably, Zhang et al. (2025) directly confirmed that toxicant exposure induces GSH depletion, which disrupts antioxidant defense and exacerbates inflammatory damage, providing critical support for our inference (Zhang et al., 2025). Together, these processes may lead to the dose-dependent accumulation of 20-COOH-LTB_4_, suggesting it may serve as a potential biomarker reflecting the intensity of the MA-induced oxidative stress–inflammation injury cascade. Direct experimental validation of the LTB4 synthesis/clearance regulatory axis and its crosstalk with canonical inflammatory pathways (e.g., MAPK/NF-κB) remains to be conducted.

To translate the potential of these two biomarkers into clinical practice, their detection feasibility warrants brief discussion. The candidate biomarkers 20-COOH-LTB4 and SAH were quantified by LC-MS/MS in the present study, a technique with prominent advantages of high quantitative accuracy, strong specificity, and high sensitivity for trace metabolite detection in complex biological samples. However, LC-MS/MS has inherent limitations for routine clinical or field application, such as high instrument cost, complex sample pretreatment, and reliance on professional technical personnel. For SAH, a mature commercial ELISA kit (e.g., CELL BIOLABS, Catalog No. MET-5151-C) directly supports its clinical translation. With a detection sensitivity of 0.2 μM, this kit features standardized operation and good reproducibility, and has been validated by multiple studies for biological sample detection, which can be quickly adapted for high-throughput clinical screening. For 20-COOH-LTB4, as a homologous metabolite of LTB4, preliminary detection can be achieved by leveraging the cross-reactivity of antibodies from existing LTB4 ELISA kits, or a dedicated ELISA kit can be developed through antibody-specific modification, exhibiting clear translational feasibility.

## 5 Conclusion

This study identifies 20-COOH-LTB4 and S-adenosylhomocysteine (SAH) as potential, dose-dependent biomarkers for methyl acetate (MA) exposure. Their excellent discriminative power was confirmed in a human cohort, where both metabolites showed complete concentration separation from blank controls and methanol (ME)-exposed groups. Mechanistically, SAH accumulation originates from the disrupted crosstalk among the TCA cycle, one-carbon metabolism, and oxidative stress pathways. In parallel, the elevation of 20-COOH-LTB4 reflects the activation of oxidative stress–inflammatory cascades induced by MA. Furthermore, MA exposure triggers systemic metabolic disturbances, encompassing mitochondrial energy crisis, purine metabolism dysregulation, and impairment of the oxidative defense system. Together, these findings provide a comprehensive mechanistic understanding of MA intoxication.

## 6 Limitations

Several limitations in this study should be acknowledged. First, the sample size was relatively small in both the animal experiment and human cohort. Some rats in the MMA and HMA groups were excluded due to accidental death during the experiment, which further reduced the final number of animals included in statistical analysis. Although the results were statistically significant, the small sample size may increase the risk of false positives and potential overfitting. Therefore, the perfect diagnostic performance with an AUC value of 1.00 should be interpreted with caution, and further validation in a larger independent population is still warranted.

## Data Availability

The datasets presented in this study can be found in online repositories. The names of the repository/repositories and accession number(s) can be found below: https://ngdc.cncb.ac.cn/omix/preview/ER43qXrf, no.OMIX014564.
