# Supplementary material for: Oxidative stress mechanisms and potential biomarkers of methyl acetate poisoning: a urinary metabolomics study in rat model and human occupational cohort
Source: Front Mol Biosci. 2026 Apr 17;13:1801734. doi: 10.3389/fmolb.2026.1801734 (PMC13132698; doi:10.3389/fmolb.2026.1801734)

Supplementary Material

**Table S2.** The complete list of identified differential metabolites in rats and humans

| **Metabolite of Rat** | | |
| --- | --- | --- |
| **Metabolite** | **KEGG ID** | **Log2 FC** |
| Ethanol | C00469 | -1.25 |
| Ethylene glycol | C01380 | 4.00 |
| Methylglyoxal | C00546 | 4.00 |
| 5-Aminoimidazole | C05239 | -1.67 |
| (R)-Lactate | C00256 | -4.00 |
| 5-Aminolevulinate | C00430 | -2.24 |
| D-erythro-3-Hydroxyaspartate | C19838 | -1.02 |
| Succinate | C00042 | 4.00 |
| L-Homoserine | C00263 | -2.11 |
| D-Serine | C00740 | -1.81 |
| 8-Hydroxyadenine | C22499 | 1.80 |
| gamma-L-Glutamyl-L-2-aminobutyrate | C21015 | -1.25 |
| 5'-Butyrylphosphoinosine | C06435 | 1.73 |
| O-Acetyl-L-homoserine | C01077 | -1.63 |
| Adenine | C00147 | 1.39 |
| L-Aspartate | C00049 | -1.71 |
| S-Glutathionyl-L-cysteine | C05526 | -1.90 |
| 2,3-Diketo-5-methylthiopentyl-1-phosphate | C15650 | 4.00 |
| Deoxyadenosine | C00559 | -1.37 |
| dAMP | C00360 | -2.00 |
| L-Glutamine | C00064 | -1.86 |
| cis-Aconitate | C00417 | 1.52 |
| 3-Sulfinylpyruvate | C05527 | 4.00 |
| Hypoxanthine | C00262 | 2.34 |
| Dihydrofolate | C00415 | 1.34 |
| Ectoine | C06231 | -1.09 |
| Glycine | C00037 | -1.29 |
| 5-Oxoproline | C01879 | 4.00 |
| Dihydroxyfumarate | C00975 | 4.00 |
| 2-Hydroxy-3-oxoadipate | C03217 | 4.00 |
| S-Methyl-1-thio-D-xylulose 5-phosphate | C22359 | 1.79 |
| L-Cysteine | C00097 | -1.70 |
| S-Sulfo-L-cysteine | C05824 | 4.00 |
| Ophthalmate | C21016 | 4.00 |
| 2-Propylmalate | C05994 | -1.11 |
| Arbutin 6-phosphate | C06187 | 1.74 |
| O-Phospho-L-homoserine | C01102 | -1.18 |
| L-erythro-3-Methylmalyl-CoA | C06027 | 4.00 |
| Adenosine | C00212 | 7.49 |
| (R)-2-Ethylmalate | C02488 | 4.00 |
|  | | |
| **Metabolite of Human** | | |
| **Metabolite** | **KEGG ID** | **Log2 FC** |
| Sarcosine | C00213 | 4.00 |
| S-Adenosyl-L-homocysteine | C00021 | 2.51 |
| N6-Succino-2-amino-2'-deoxyadenylate | C22395 | 4.00 |
| Leukotriene E4 | C05952 | 1.10 |
| L-Cystathionine | C02291 | -4.52 |
| Dehydroalanine | C02218 | 4.00 |
| Cys-Gly | C01419 | 4.49 |
| Creatine | C00300 | -1.98 |
| 5-Oxoproline | C01879 | 1.01 |
| 2-Oxobutanoate | C00109 | 1.49 |
| 20-COOH-Leukotriene B4 | C05950 | 2.26 |
| 2,8-Dihydroxyadenine | C22500 | 1.33 |
| 1-(5'-Phosphoribosyl)-5-amino-4-imidazolecarboxamide | C04677 | 2.52 |
| Homocitrate | C01251 | 4.00 |
| (R)-2-Ethylmalate | C02488 | 3.13 |
| 15-Keto-prostaglandin I2 | C04835 | 4.00 |

**Figure S1.** Detailed metabolic changes and differential metabolites supporting MA‑induced core metabolic disturbance modules.

The legend of Figure S1: Colored blocks represent the four key metabolic disturbance modules consistent with Figure 9. Internal rectangular boxes indicate specific abnormal events, which are supported by the corresponding differential metabolites shown below. Arrows indicate the regulatory relationships among these differential metabolites. The four panels in each heatmap next to nodes represent the metabolite levels in rat Con, LMA, MMA and HMA groups (log2-normalized peak intensities), while the two panels represent those in the two human groups (log2-normalized peak intensities).


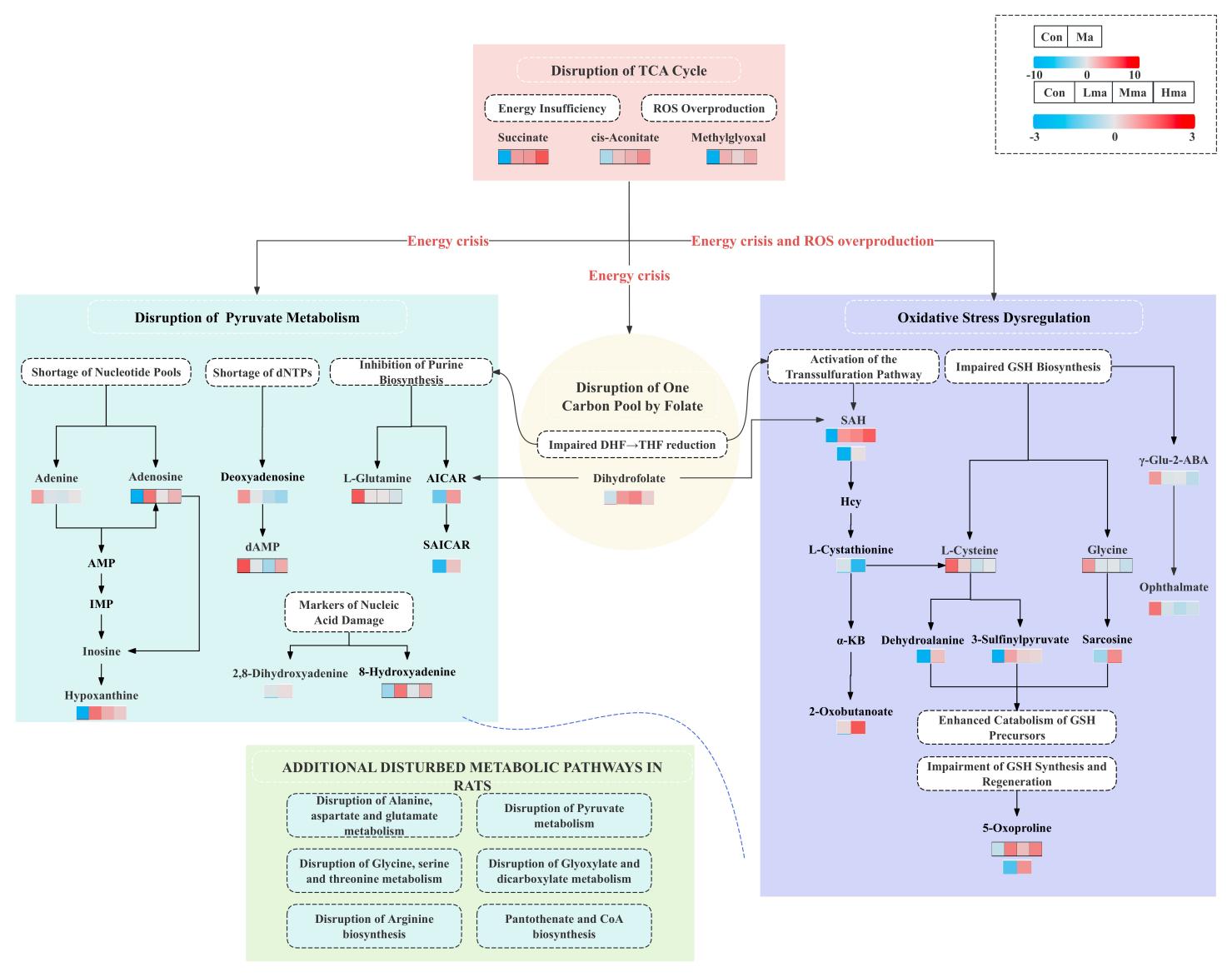

Supplement: Supplementary file 1 [file Supplementaryfile1.docx]
